# Avolition as the core negative symptom in schizophrenia: relevance to pharmacological treatment development

**DOI:** 10.1038/s41537-021-00145-4

**Published:** 2021-02-26

**Authors:** Gregory P. Strauss, Lisa A. Bartolomeo, Philip D. Harvey

**Affiliations:** 1 Department of Psychology, University of Georgia, Athens, GA USA; 2 Department of Psychiatry and Behavioral Sciences, University of Miami Miller School of Medicine, Miami, FL USA

**Keywords:** Schizophrenia, Psychology

## Abstract

Negative symptoms have long been considered a core component of schizophrenia. Modern conceptualizations of the structure of negative symptoms posit that there are at least two broad dimensions (motivation and pleasure and diminished expression) or perhaps five separable domains (avolition, anhedonia, asociality, blunted affect, alogia). The current review synthesizes a body of emerging research indicating that avolition may have a special place among these dimensions, as it is generally associated with poorer outcomes and may have distinct neurobiological mechanisms. Network analytic findings also indicate that avolition is highly central and interconnected with the other negative symptom domains in schizophrenia, and successfully remediating avolition results in global improvement in the entire constellation of negative symptoms. Avolition may therefore reflect the most critical treatment target within the negative symptom construct. Implications for targeted treatment development and clinical trial design are discussed.

## Overview

Negative symptoms were initially described by Kraepelin and Bleuler, the founders of the modern schizophrenia construct^1–3^. Kraepelin defined schizophrenia or “dementia praecox” as an illness with a progressively deteriorating course across multiple features starting in the late teens or early adulthood. Less known, but equally central, is the role Kraepelin attributed to avolition in its vital impact on the manifestation of schizophrenia*, “*a weakening of those emotional activities which permanently form the mainsprings of volition…“^2,3^. Similarly, Bleuler pointed out that “indifference seems to be the external sign of their state…The will…disturbed in a number of ways, but above all by the breakdown of the emotions…The patients appear lazy and negligent because they no longer have the urge to do anything either of their own initiative or at the bidding of another.*“*^1^. Although conceptualizations of negative symptoms have been refined over the years^4^, reduced motivation and diminished emotional expression are still considered core facets of the illness^5,6^.

The introduction of DSM classification in the 1950s and 1960s was an attempt to improve communication between mental health professionals and achieve reliability among clinicians and researchers^7^. With the focus on reliability of measurement central to successive modifications of the diagnostic systems (Feigher Criteria, Research Diagnostic Criteria, DSM-III^8^), it is natural that much of the focus was on positive symptoms. Positive symptoms, and especially first-rank symptoms, such as thought insertion, withdrawal, and broadcasting, or delusions of control, can be more reliably reported, observed, quantified and communicated than negative symptoms. The rating scales used to collect diagnostic information such as the Current and Past Psychopathology Scales (CAPPS^9^), the Schedule for Affective Disorders and Schizophrenia (SADS^10^), and eventually the Structured Clinical Interview for the DSM (SCID), featured detailed descriptions of multiple positive symptoms and a deemphasis on the assessment of negative symptoms. For instance, the 2007 edition of the SCID, developed for the DSM-IV-TR edition^11^, assesses 15 different delusions, 8 different hallucinations, and 3 negative symptoms: Avolition, Alogia, and Affective flattening. The availability of antipsychotic drugs, which ameliorate positive symptoms, but worsen or do not improve negative symptoms, has further highlighted the role of positive symptoms.

Nevertheless, researchers and clinicians rapidly realized that negative symptoms, a core feature of schizophrenia and the main contributor to the disease’s burden^12–16^, remained unaddressed by antipsychotic drugs^17,18^. Currently, no medication has received an indication for negative symptoms from the United States Food and Drug Administration and there is no recommended pharmacological treatment for negative symptoms in schizophrenia^19^. This awareness triggered a renewed interest in developing targeted treatments for negative symptoms^4^.

## The current measurement of negative symptoms: instruments and their properties

Simply viewed, negative symptoms encompass *all that is missing* from the normal range of emotions and behaviors. The definitions of negative symptoms and the related measurement scales are broad aggregates of examiner observations and examinee reports. Central to this challenge is the lack of a clear definition of what is “the normal range” of these behaviors. While the extremes of normality and abnormality in this domain can be identified with ease, the extent to which more moderate behaviors are quantified, defining incremental levels of impairment and a clear threshold for separation from “normal”, is challenging. Further, despite active research in the field, no objective bio-markers to diagnose or quantify negative symptoms are currently available. Therefore, what is observed and reported is unavoidably affected by subjective interpretations, raising questions about validity and reliability of the assessments of this construct^20–22^.

The first attempts to quantify negative symptoms were the Brief Psychiatric Rating Scale (BPRS^23^) Scale for the Assessment of Negative Symptoms (SANS^24^), Positive and Negative Syndrome Scale (PANSS^25^), and Negative Symptom Assessment^26^. However, these first-generation scales have been criticized in recent years due to outdated item content, conceptual limitations, overreliance on behavioral indicators at the expense of experiential factors, and psychometric issues^20^.

As an example of issues associated with outdated item content and conceptual limitations, researchers who factor-analyzed the PANSS criticized the content of the original PANSS negative symptoms subscale as including items which, on the one hand, conceptually should not have been designated as negative symptoms, and on the other hand, misclassified negative symptoms under the general symptom’s subscale^27^. Furthermore, both the PANSS and the SANS contain items that assess cognitive functions and disorganization rather than negative symptoms^20^. Finally, the PANSS item assessing “active social avoidance” (G16), is clustered among the general psychopathology subscale items in the original scale^25^, and is found to be statistically related to other negative symptoms, as evidenced by its repeated loading with other negative symptoms in factor analyses of the PANSS^28–30^. However, according to the last edition of the PANSS Manual, G16 should assess social withdrawal due to fear, hostility, and distrust. Therefore, it clearly seems to be defined as a secondary negative symptom^31^ and possibly should not be used as an indicator of asociality.

Overreliance on potentially multi-determined behavioral indicators is apparent in the SANS and PANSS, which focus on the behavioral/functional consequences of avolition-apathy, but not the subjective, internal experience and emotional aspect of avolition, understood to be the very core of the schizophrenic illness^32^. The SANS, for instance, defines several elements of real-world functioning as negative symptoms, rather than as a potential behavioral consequence of these symptoms (e.g., impersistence in work and school). Because a patient’s behavior and functioning can be affected by many factors external to the illness, such as poverty, social exclusion, poor education, and limited work and social opportunities, assessments made predominantly on observed behavior and function (although reliable) may lead to a biased assessment of the severity of negative symptoms in general, and of avolition in particular. There are other illness-related factors such as delusions and hallucinations that lead to episodic reductions in functional behaviors as well. Finally, it is critical for valid ratings to disentangle primary, illness-related, negative symptoms and secondary negative symptoms, which can be related to other illness-related variables, social factors, or concurrent pharmacological factors. This process, while critical, is often very difficult and requires both separation of subjective experiences from actual behaviors and longitudinal considerations of the influence of psychosis on apparent negative symptoms.

These limitations and uncertainties have spurred debate around the definition of negative symptoms and given rise to the second-generation of measurement instruments attempting to address limitations of the previous scales^4,33^. These scales include the Clinical Assessment Interview for Negative Symptoms (CAINS^34^) and Brief Negative Symptoms Scale (BNSS^35^). Both measures were designed to assess the 5 core negative symptom domains delineated in the 2005 NIMH Consensus Development Conference (anhedonia, avolition, asociality, alogia, blunted affect) according to modern conceptualizations^4^. Psychometric properties of the CAINS and BNSS have been excellent for English and translated versions (e.g., test-retest reliability, internal consistency, inter-rater agreement, convergent validity, discriminant validity)^34,36^. Sensitivity to change, for both total and domain scores, has been demonstrated in clinical trials^37,38^. To further improve the assessment of negative symptoms and obtain longitudinal rather than cross-sectional data, a third-generation of measurement tools, based on digital phenotyping (i.e., the use of mobile devices, such as smartphones and smartbands, to initiate data collection in everyday life) is currently under development. Both active (e.g., ecological momentary assessment surveys, ambulatory videos) and passive (e.g., geolocation, accelerometry, acoustic measures) digital phenotyping measures may hold promise for measuring negative symptoms more objectively in the context of everyday life^39–42^.

Based on a consensus delineated in the National Institute of Mental Health Consensus Development Conference^4^, and on factor analysis of the assessment scales, the current concept of negative symptoms encompasses the following domains:

1. Blunted affect: a decrease in the outward expression of emotion in relation to facial expression, vocal expression, and body gestures.
2. Alogia: a reduction in the quantity of speech or amount of spontaneous elaboration.
3. Anhedonia: reductions in the intensity and/or frequency of pleasurable experiences across activity domains (e.g., social, physical, recreational, work/school).
4. Asociality: a reduction in the frequency of social interaction and interest in forming close relationships with others.
5. Avolition: a reduction in the initiation of and persistence in goal-directed activities, and the desire to perform such activities. It is both a subjective reduction in interests and desires (internal experience) and a behavioral reduction of self-initiated and purposeful acts, which should only be considered negative symptoms if the behavior is a direct consequence of the internal state.

Initial exploratory factor analytic studies of the PANSS, SANS, CAINS, and BNSS indicated that these 5 domains produce a 2-factor structure, reflecting diminished expression (EXP) and motivation and pleasure (MAP)^5,6,32,34,36,43^. More recent confirmatory factor analyses and network analyses indicate that negative symptoms are best conceptualized in relation to either 5 discrete domains (anhedonia, avolition, asociality, blunted affect, alogia) or a hierarchical structure consisting of two overarching higher-order dimensions (MAP and EXP) that have more basic subordinate domains (MAP = anhedonia, avolition, asociality; EXP = blunted affect, alogia)^32,37,43–46^. Thus, both two dimension and five domain conceptualizations of negative symptoms are highly relevant^32^ and are in no way mutually exclusive.

In summary, older scales, such as the PANSS and its sub-scales, although still widely used in research and drug development, are not ideal for assessing negative symptoms as currently conceptualized. Newer scales, which already have good validation data, such as the BNSS and its respective subscales and domains, may be more appropriate for the evaluation of pharmacological treatments targeting negative symptoms or specific elements of negative symptoms, such as avolition, because they capture both manifestations of the symptom, internal motivation, and real-world behavior^37^, rather than just the observable behavioral dimension, as is the case with PANSS.

## The concept of avolition and its central role in negative symptoms of schizophrenia

Avolition reflects the decrease in the motivation to initiate and perform purposeful activities^27,47^. In the larger picture, the individual seems to experience a lack of interest in improving themselves intellectually, physically, socially, and financially. The activities that are not performed range from elementary ones, such as grooming, personal hygiene, or preparing food, and extending to more complex acts, such as going to work and/or school and engaging in social activities. A person experiencing avolition may stay at home, staring at the TV for hours and days, hardly following the content, rather than seek work or peer interaction. In fact, EMA surveys of participants with schizophrenia who are rated as having avolition often find that they are home and alone for the majority of the day^48^ and that they are more likely than healthy people to have only engaged in one, typically inactive, activity in the past hour^12^. Differently from a person experiencing a major depression associated with unipolar or bipolar disorder, a person experiencing avolition would not necessarily complain of depressed mood, insomnia, guilt, or suicidal thoughts. Even anxious mood seems absent in people experiencing avolition, suggesting a general experiential emptiness.

Occasionally, avolition can manifest in an individual who does not visibly meet DSM/ICD criteria for mental illness^49^. However, in most cases, avolition is an underlying symptom of a complex mental illness^50^. The most common and severe manifestation of avolition occurs in schizophrenia and schizophrenia-spectrum disorder. Still, it has also been described in autism and all ranges of affective and personality disorders^50^.

Avolition is common in first-episode schizophrenia cases^51^, and in even higher proportion in individuals at high risk for schizophrenia^52^, regardless of whether they develop the full syndrome of schizophrenia or not. Avolition is the strongest and most reliable predictor of certain elements of functional outcomes^13,53–56^. Furthermore, lack of motivation has been found to mediate the relationship between cognitive impairment and poor social outcomes^57^. Avolition seems to be associated with poor premorbid social adjustment during childhood, insidious onset of psychosis, deficits in executive functioning and abstraction-flexibility, and male gender^58,59^.

Avolition, apathy, anhedonia and amotivation, have created a nomenclature conundrum which reflects both a certain degree of phenomenological overlap and a likely pathophysiologic interconnection mediated by dysfunctional motivational circuit^21,60,61^. Motivation is a multi-dimensional construct, affecting hedonic experience, reward prediction, reward valuation, and effort valuation. This is the framework adopted by the positive valence system of the Research Domain Criteria (RDoC)^62^. It has been postulated that abnormalities in reward functions affect motivation in schizophrenia^61,63^. Apparently, patients with schizophrenia experience levels of pleasure similar to healthy controls when engaging in pleasant activities^37^. However, they less frequently engage in behaviors aimed at obtaining rewards and goal-directed outcomes because they have decreased capacity to anticipate future rewards^21^. Deficits in other aspects of reward processing may also play a role in preventing intact hedonic responses from influencing decision-making abilities needed to initiate goal-directed activity, such as effort-cost computation, reinforcement learning, and value representation^61,64–66^.

The central role of avolition in schizophrenia patients with negative symptoms is supported by network analysis^37^. Network analysis is used to assess complex systems, focusing on the interrelatedness of system components. The idea behind this approach is that psychiatric disorders emerge from the interactions among symptoms in a network; hence the presence of one symptom increases the probability that a related set of symptoms will also manifest. Symptoms are viewed in terms of their density, and dense networks are closely interconnected and co-activate once symptoms exacerbate, forming closely joined clusters of psychopathologies that support each other and become self-sustaining. Often, an individual symptom, avolition for example, will be more central than others in a network, with strong interconnections to other symptoms that cause those symptoms to emerge whenever the central symptom is manifested. In tightly connected networks, the activation of a central symptom may lead to the activation of other symptoms, even after the factors that triggered it had disappeared. This process may account for the symptom’s chronicity. Rather than examine each symptom individually, as a single effect of a causal disorder, network analysis considers the interaction between each symptom and all the other ones. For example, it is plausible that avolition is also a determinant of decreased speech output (alogia), reductions in facial and vocal expression of emotion (blunted affect), slow movements, diminished pursuit of pleasurable activities or the ability to enjoy them (anhedonia), and limited engagement in social interactions (asociality) due to a common amotivational substrate. The ability to engage in these behaviors may be preserved, but the level of motivation needed for behavioral initiation may not cross the threshold required to execute them^61,67^. Therefore, pharmacological agents which can improve a central symptom such as lack of motivation may result in the overall improvement of negative symptoms^37^.

Rating scales of avolition mostly include a retrospective assessment, which combines several sources of information. In the SANS, apathy/avolition is assessed by three items, exclusively focusing on the subject’s behavior: grooming, persistence at work/school, and physical anergia. In the PANSS, two items assess avolition: emotional withdrawal (N2) and passive apathetic social withdrawal (N4), both of which rely upon caregiver’s report on the patient’s interest and emotional involvement in daily activities. The BNSS includes two distinct items for avolition, internal experience, and avolition behavior; both items of these items cover motivation for work/school, recreational activity, self-care, and general time spent in inactivity. The CAINS uses two items to assess avolition: motivation to work or attend school and motivation for recreational activities. The relative weight that should be assigned in rating avolition to the patient’s report versus the caregiver’s report is still a matter of debate among psychometricians^68^.

Similar to negative symptoms in general, attributing passively or actively originating reductions in social engagement only to avolition may be problematic, because reduced social engagement can be determined by multiple factors other than avolition. There are clearly demonstrated connections between lack of opportunity (related to social and economic deprivation), lack of social skills, impairments in social cognition, rejection related to family conflicts, exclusion related to stigma, or preoccupation with delusions and hallucination. These factors have origins in society, the individual, their families, and the other features of illness. All these factors can contribute to observations of impoverished social networks, yet these factors are distinct from social or occupational failures that derive from avolition. Thus, although reliance on behavioral observations may be more accurate than self-reports^69^, both can lead to erroneous ratings on existing scales, for reasons that do not reflect avolition^15,70^.

As there is a phenomenological and functional overlap between avolition, anhedonia, and apathy, the question of whether there is a separate biological mechanism for avolition is still under investigation. Studies have proposed that blunted dorsal striatal activity might be specifically related to avolition (motivation) and not to anhedonia (pleasure) and could be a neural correlate of impaired action-selection^63^. Avolition and anhedonia aspects of apathy can be mapped onto functional cortico-striatal pathways. Two possible mechanisms have been proposed: a) a dysfunctional motivational value circuit related to (anticipatory) anhedonia, and b) a dysfunctional motivational salience circuit related to avolition (lack of motivation)^15^. Regarding a trans-diagnostic understanding of these distinct mechanisms, it is also relevant to investigate the overlap and differentiation of apathy, anhedonia, and depression. Apathy and depression occur trans-diagnostically in neurological and psychiatric disorders. Anhedonia is a symptom of apathy in schizophrenia, but also a core symptom of depression^71^. It has been suggested that the overlap between anhedonia and apathy in schizophrenia can be explained by specific deficits in anticipatory anhedonia, while consummatory hedonic experiences are relatively intact^21,72^. Similarly, it is a common phenomenological complaint among people experiencing major depression that the consummatory element of the anhedonia sequence is actually impaired: “Nothing tastes as good or feels as good.” Recent research has suggested that major depression may be associated with both anticipatory and consummatory anhedonia^72^. Trans-diagnostic multi-component frameworks of apathy and anhedonia might help to identify shared and distinct behavioral, computational, and neuroimaging parameters of both conditions—whether motivational (avolition) and hedonic (anhedonia) aspects of apathy can be mapped onto a distinct functional cortico-striatal pathway.

## Avolition and drug development

After two decades of psychotropic drug development with moderately superior tolerability profile over the previous drugs, the 2000s have been characterized by stagnation of productivity in psychiatric drug development^73,74^. The stagnation has been attributed to a lack of pathophysiological understanding of the mental disorders, difficulties in defining targets, poor translation from animal models to human, and the absence of biological markers^74^. Adherence to diagnostic systems that were not specifically designed for drug development, such as the DSM, and the use of overinclusive outcome measures and scales has also been suggested as a major reason for the failure to develop novel psychotropics^75–77^.

In this regard, treatment trials targeting negative symptoms in schizophrenia are not different in that they enroll patients whose brain dysfunctions are probably caused by diverse, heterogenous biological underpinnings. For example, a 20-year-old participant in a work rehabilitation program with poor premorbid adjustment, with an IQ of 85, persistent but low-intensity paranoid ideation, active social withdrawal, and severe alogia differs from a 35-year-old formerly practicing lawyer with an IQ of 120 in the 8th month of remission from what has been the second episode of acute psychosis, who is currently incapacitated by severe avolition, reduced facial expression, and restricted vocal inflection. Nevertheless, both would probably qualify for a trial targeting negative symptoms in schizophrenia^78^.

Indeed, it is accepted by academicians, clinicians, and regulators that the behavioral syndromes defined by DSM classifications and the assessment scales attempting to define the severity of these impairments do not correspond to specific, putative biological processes and brain abnormalities. Nevertheless, meeting criteria for the DSM classifications and manifesting a threshold level of severity still constitute the criteria for patients’ inclusion in trials and the basis for independent investigator-initiated studies and new drug applications.

While the solution depends on progress in understanding normal and abnormal brain functioning, the general idea is to reconsider the relationship between psychiatric disorders and drug development in the following manner: (a) identify basic symptoms that manifest trans-diagnostically across DSM syndromes; (b) identify biological circuits or genetic-molecular processes and markers common to these manifestations; (c) design and utilize inclusions and outcome severity scales focused on narrow, easily definable symptoms; and (d) devise therapeutic interventions that engage targets common to these circuits and/or genetic-molecular processes.

Concurrently, it is important as well to determine the breadth of targeted symptoms. Depending on the rating scale, negative symptoms have at least two and probably 5 dimensions that are separable from each other in terms of evaluation of the presence and severity of the features. As noted above in the discussion of network analysis, some of the negative symptoms features may be central and others may be determined by the presence and level of severity of the central features. Thus, the peripheral features are likely driven both the central features and other factors. The other factors, as described above, may have nothing to do directly with psychiatric illnesses. In a sense, treatment of the peripheral features would be much more challenging, and much less likely to result in functional gains than treatment of central features. Conversely, targeting central features identified using network approaches may be a more efficient approach that yields widespread improvement in negative symptoms and, in turn, improved functioning.

RDoC, the initiative of the US National Institute of Mental Health (NIMH), is such an approach of defining critical dimensions or domains of functioning that are smaller than syndromes, not necessarily pathological on their own, and definable with greater levels of specificity. It proposes to divide current DSM syndromes into simpler, more basic, observable manifestations for which measurable physiological correlates can be devised^79^. The assumption is that observable behaviors and emotions and their respective neurobiological measurable correlates span multiple DSM disorders^80^ and may be combined with each other in order to produce the larger syndromes^74^. However, if the larger syndrome cannot be produced when the smaller domain is absent, then treatment of these smaller domains may have the potential to reduce morbidity in ways that are equivalent to the treatment of the larger syndrome.

An initiative similar to the RDoC is the Psychiatric Ratings using Intermediate Stratified Markers (PRISM). It is a European Union-funded initiative intending to investigate the common and the distinct biological background of reduced social engagement in schizophrenia, major depressive disorder (MDD), and Alzheimer disease (AD), and to identify biological targets that will inform the development of drugs for avolition and reduced social engagement across syndromes^74^. However, reduced social engagement is nevertheless a complex behavior that can be modulated by aging, concomitant medical or neuropsychiatric conditions, social, vocational, and economic circumstances. A more basic component of reduced social engagement is disturbance in motivation or avolition which, like the larger construct of reduced social engagement, is manifested in stroke^81^, traumatic brain injury^82^, schizophrenia, and MDD^83^. Looking beyond the restrictions of the DSM system, it might be possible to develop a drug to treat these conditions trans-diagnostically. Unfortunately, previous attempts to target neurochemical systems that have been implicated across these conditions have generally been unsuccessful^84–87^. Since most of these drugs have primarily targeted dopaminergic mechanisms, exploration of non-dopaminergic targets may be beneficial, as there has been some recent evidence for success with drugs achieving their action via non-dopaminergic mechanisms^88–90^. Since such drugs have been shown to achieve their effects by impacting avolition from a network perspective, focusing on this most central component of negative symptoms may be key to observing widespread improvements in negative symptoms^37^.

## Conclusions

The current trend to identify basic symptoms that manifest trans-diagnostically, investigate biological circuits and markers common to these manifestations, and to develop drugs that engage targets common to these trans-diagnostic circuits and/or genetic-molecular methods should be encouraged.
